# The distinct RNA-interaction modes of a small ZnF domain underlay TUT4(7) diverse action in miRNA regulation

**DOI:** 10.1080/15476286.2021.1991169

**Published:** 2021-11-01

**Authors:** Belén Chaves-Arquero, Katherine M. Collins, Evangelos Christodoulou, Giuseppe Nicastro, Stephen R. Martin, Andres Ramos

**Affiliations:** a Institute of Structural and Molecular Biology (ISMB) instead of (Ismb), University College London, London, UK; b Structural Biology Science Technology Platform, The Francis Crick Institute, London, UK; c Macromolecular Structure Laboratory, The Francis Crick Institute, London, UK

**Keywords:** TUT4/zcchc11, pre-let7i miRNA, ssRNA recognition, nuclear magnetic resonance, Biolayer Interferometry

## Abstract

TUT4 and the closely related TUT7 are non-templated poly(U) polymerases required at different stages of development, and their mis-regulation or mutation has been linked to important cancer pathologies. While TUT4(7) interaction with its pre-miRNA targets has been characterized in detail, the molecular bases of the broader target recognition process are unclear. Here, we examine RNA binding by the ZnF domains of the protein. We show that TUT4(7) ZnF2 contains two distinct RNA binding surfaces that are used in the interaction with different RNA nucleobases in different targets, i.e that this small domain encodes diversity in TUT4(7) selectivity and molecular function. Interestingly and unlike other well-characterized CCHC ZnFs, ZnF2 is not physically coupled to the flanking ZnF3 and acts independently in miRNA recognition, while the remaining CCHC ZnF of TUT4(7), ZnF1, has lost its intrinsic RNA binding capability. Together, our data suggest that the ZnFs of TUT4(7) are independent units for RNA and, possibly, protein-protein interactions that underlay the protein’s functional flexibility and are likely to play an important role in building its interaction network.

## Introduction

TUT4/Zcchc11 (zinc-finger, CCHC domain-containing protein 11) is a non-templated U-specific polymerase that, together with the related and partially redundant protein TUT7, plays a key role in organismal development. TUT4(7) is essential for oocyte development [1], the maternal-to-zygotic transition [2] and the later development of the embryo, among others [3,4]. Not surprisingly, dis-regulation of TUT4(7) is linked to a large set of cancer pathologies [3,5,6].

TUT4(7) plays important roles in different stages in development and in adults these are linked to its uridylation of a broad range of RNA targets that differ in structure and sequence, including mRNAs [7–9] but also miRNA precursors [10–15] and mature miRNAs [16–20]. In some settings, TUT4(7) binding leads to 3ʹ poly(U)ridylation and subsequent RNA degradation. In others, TUT4(7) mediates the addition of a single U that stabilizes the target RNA.

**Figure 1.**
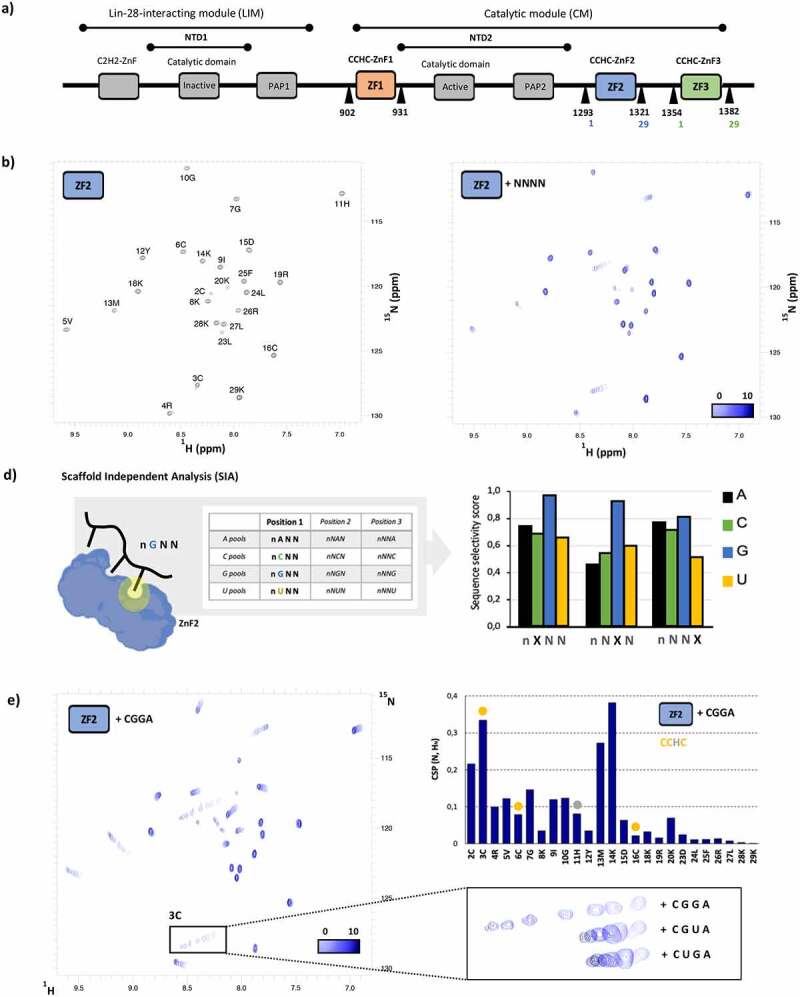
A) Domain structure of hTUT4. The three CCHC ZnF domains are coloured in Orange, blue and green. the boundaries of the ZnF1, ZnF2 and ZnF3 constructs used in this study are reported using both the full-length hTUT4 amino acid count (black) and, for clarity, the amino acid count of that we use in this manuscript (colours). please note that the ZnF2-3 construct starts at the N-terminal boundary of ZnF2 and ends and the C-terminal boundary of ZnF3. b) [^15^N ^1^H] HSQC spectra of CCHC-ZnF2. c) O verlay of the [^15^N ^1^H] HSQC spectra of ZnF2 with NNNN RNA at ratios from 1 to 0 (light blue) to 1 to 10 (dark blue). d) S caffold Independent Analysis (SIA) of the hTUT4 ZnF2. left: SIA determines the nucleobase preference of a domain in any wanted position of the bound sequence by comparing the interaction of the domain with four quasi-degenerate RNA sequences, each with one of the four nucleobases in the position to be scanned. A workflow and representative data for this assay are found in Supplementary Figure 1. right: SIA scores for hTUT4 ZnF2. e) l.eft: overlay of [^15^N ^1^H] HSQC spectra recorded on ZnF2 with CGGA at protein to RNA ratios from 1 to 0 (light blue) to 1 to 10 (dark blue). I. nset: zoom of the Cys 3 amide resonance. In the inset, the corresponding peaks from equivalent titrations with the CGUA, CUGA RNAs are also displayed. please note that peaks from the other two titrations have been shifted in order to minimize overlap and better appreciate differences in saturation and linewidth. Top right: CSP measured in the CGGA titration plotted against the protein sequence

The best studied function of TUT4(7) is the regulation of the biogenesis of the tumour suppressor Let-7 miRNA. TUT4(7) acts in combination with the protein adaptor Lin28 to recognize and poly(U)ridylate the Let-7 miRNA precursor, leading to its degradation by the exonuclease DIS3L2 [10,(11,12]. TUT4-mediated poly(U)ridylation of pre-Let-7 requires the formation of a three-component pre-let-7-Lin28-TUT4(7) complex, where TUT4(7) interacts with both the Lin28 protein and the pre-Let-7 miRNA. Recent structural and molecular work on the TUT4 and TUT7 proteins has provided an insight into this interaction but has also suggested that one of the ZnF domains of TUT4(7) makes direct contact with the polyU, and facilitates processive poly-uridylation [21]. hTUT4(7) contains an amino-terminal domain, also known as the Lin-28 interacting module (LIM), and a carboxy terminal domain or catalytic module (CM) (Figure 1(a)). The CM module comprises a catalytically active domain (NTD2) sandwiched between two CCHC ZnFs (ZnF1 and 2) followed by a low sequence complexity region that includes a third ZnF (ZnF3). The CM interacts with the terminal base pair and the 3’single stranded end of the RNA molecule (7). Importantly, the second ZnF of the CM (ZnF2) makes contacts with the O2 and O4 moieties of a Uridine in position U2 of a single stranded poly U RNA. This specific contact has been proposed to stabilize the nascent poly(U) chain and to promote efficient poly(U) tailing [21,22].

In addition to recognizing pre-miRNA, hTUT4(7) has been shown to interact with and regulate poly-uridylate mRNAs(7) [1,2,8,9], mature miRNAs [16,17,18–20] and other ncRNAs [23,24]. Interestingly, one of the miRNAs regulated by TUT4(7) is the tumour suppressor Let-7i, and TUT4(7) has been shown to regulate Let-7i mono-uridylation and Hox gene regulation during development.

While structural and molecular work from a number of groups has provided an account of TUT4(7) interactions with the hLin28 protein and the Let-7 pre-miRNA target, we have a limited understanding of how hTUT4(7) recognizes the diverse set of cognate RNAs. It seems likely that the single-stranded, sequence specific ZnF domains play a key role in this recognition. ZnF2 has been shown to recognize RNA in the poly-Uridylated targets in a very specific structural setting, but how ZnF1 and ZnF3 interact with the RNA targets is unclear. In this work, we ask what is the role of TUT4(7) ZnF domains in RNA recognition.

## Results

### ZnF2 folds as an independent structural unit that recognizes specific RNA sequences

A recent crystal structure has shown that the second ZnF domain of hTUT7 makes specific contacts with a U within a UU dinucleotide, and it has been proposed that the domain stabilizes the poly(U) tail and increases processivity in pre-Let-7 poly-Uridylation. However, it is not clear whether this domain, which flanks the NTD2 catalytic domain, plays a wider role in recognizing and recruiting different RNA targets. Interestingly, ZnF2 directly engages in RNA recognition when NTD2 is bound to the UU di-nucleotide but not when a U1-derived RNA is bound instead. We therefore examined whether ZnF2 can fold and recognize RNA independently from NTD2, and what its base sequence specificity is. As a first step, we tested the fold and stability of the free ZnF2 using NMR. The ^15^N-correlation spectrum of the domain displays 29 well-dispersed peaks. These correspond to 29 backbone amide resonances, which is the expected number for ZnF2, and indicates that the construct folds into a single, structured, conformation in solution (Figure 1(b)). Then, we tested the sequence-independent RNA binding of ZnF2 using a randomized NNNN RNA tetramer and NMR spectroscopy. The comparison of ^15^N-correlation experiments recorded at different points of a titration showed selective changes in a set of ZnF2 amide resonances and confirmed RNA binding takes place on a specific surface (Figure 1(c)).

The resonance shifts in the NNNN titration indicate that the protein interacts with RNA with K_d_ values in the micromolar range. As NMR operates at micromolar concentrations of protein, it is ideally suited to report on the interactions. Next, we investigated *de novo* the nucleobase preference of hTUT4 ZnF2 using NMR Scaffold Independent Analysis (SIA), a method that can be used to define the nucleobase preference of isolated RNA-binding domains at each of the bound positions [25] (Supplementary Figure 1). The concept of SIA is based on comparison assays where a domain is bound to quasi-degenerate sets of RNA, with only one specific nucleobase. The binding to the different sets is examined using the shift of peaks in the NMR spectra upon addition of the RNAs, which allows one to establish how RNA sets with different nucleobases in the same position bind to RNA. The data are then normalized and averaged across different RNAs and NMR peaks [25] (Supplementary Figure 1), obtaining a series of comparative semi quantitative scores that report on the nucleobase preference of the ZnF domain (Supplementary Figure 1). In our SIA assays, we tested the potential of hTUT4 ZnF2 to recognize up to three nucleobases within a four-nucleotide RNA (we found it best to add a nucleotide at the 5ʹ to minimize boundary effects). Our analysis indicates that hTUT4 ZnF2 recognizes two Gs with significant specificity in the first and second scanned position (Figure 1(d)), while the third scanned position shows low nucleobase preference. We validated and quantified the nucleobase preference in positions one and two by comparing the binding of hTUT4 ZnF2 to the GG-containing CGGA sequence to the previously observed NNNN RNAs and to two sequences where one of the two Gs was mutated to U (CUGA and CGUA) (Figure 1(e) and Supplementary Figure 2). In our NMR experiments, the resonances are in a fast exchange regime on a chemical shift timescale. Binding of CUGA and CGUA is too weak to obtain an accurate value for the K_d_; however, comparison of the shift of peaks at equivalent protein to RNA ratios can be used as a proxy to evaluate the bound protein molar fraction. This indicated that CUGA and CGUA both interact with the protein, but with significantly lower affinity than CGGA (Supplementary Figure 2), in agreement with the SIA results (Figure 1(d)). Because the binding to CGGA RNA is tighter than to CUGA/CGUA saturation was reached in the titration. This allowed us to fit the shifts from the free to the bound position as a function of RNA concentration and obtain a K_d_ of 3.7 ± 0.4 µM for the ZnF2-CGGA complex (Supplementary Figure 3).

### ZnF2 recognizes poly(U) RNA tails and G-rich target sequences using distinct surfaces

**Figure 2.**
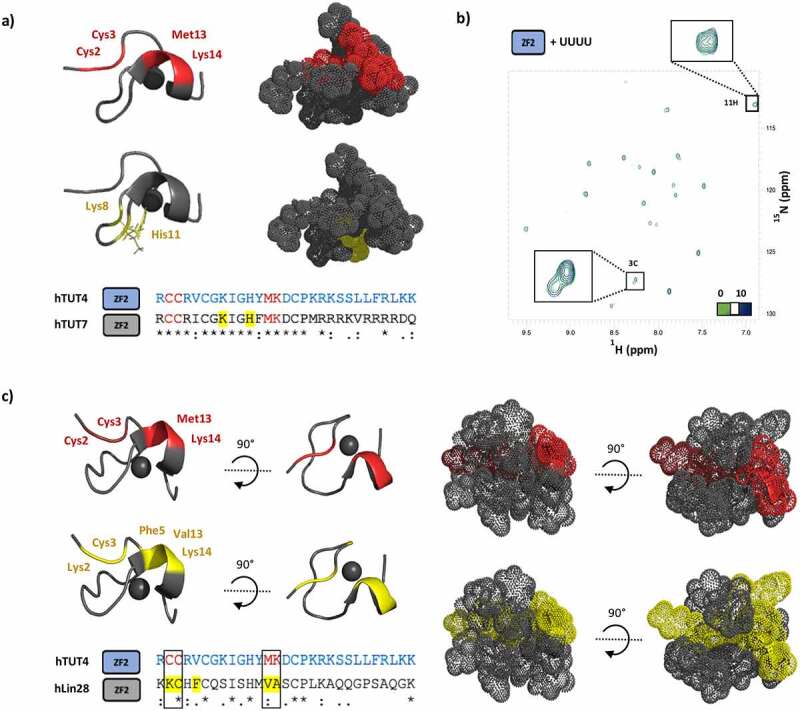
The CCHC ZnF domain binds RNA using different surfaces in different protein-RNA complexes. a) Top: The protein-RNA interaction surfaces in the hTUT4 ZnF2-CGGA (NMR) and hTUT7 ZnF2-UU (X-ray) complexes are mapped in red (top) and yellow (bottom) respectively on the structure of hTUT7, which is here displayed using both a cartoon and surface representation, for clarity of visualization. bottom: the same colour coding is used to map the interactions on the sequence alignment of hTUT4 and hTUT7 ZFs2. b) O verlay of [15 N1 H] HSQC spectra of ZnF2 with UUUU at protein to RNA ratios 1 to 0 (green) and 1 to 10 (dark blue). The region containing the Cys3 and His11 residues has been enlarged. no significant chemical shifts perturbation is observed, indicating the lack of meaningful interactions. c) Top left, and right of this panel: the protein-RNA interaction surfaces in the hTUT4 ZnF2-CGGA and hLin28 ZnF2-AGGAGAU NMR complexes were mapped in red (top) and yellow (bottom) respectively on the structure of hLin28 ZnF2, which is here displayed using both a cartoon and surface representation, for clarity of visualization. Bottom left of the panel: the same colour coding is used to map the residues on the sequence alignment of the two domains. The two residues with the largest chemical shift changes upon RNA binding are the same in the hTUT4 and hLin28 complexes and they are highlighted with a box

One important question is whether the TUT4(7) ZnF2 interacts with the CGGA RNA and the UU RNA in the TUT7 complex, using the same surface. In addition to providing information on affinity and specificity, our NMR Chemical Shift Perturbation (CSP) data allow us to define the ZnF2 surface that mediates the interaction with the CGGA RNA, provided we can assign the individual NMR cross-peaks to the amide resonances of specific amino acids. In order to do this, we assigned the backbone resonances of ZnF2 using a standard set of NMR experiments (Figure 1(b)) and mapped the amide chemical shift changes of hTUT4 on the published structure of the close homologue hTUT7. This defined a continuous surface, where the largest changes are in the backbone amides of Cys 2, Cys 3, Met 13 and Lys 14 (Figure 2(a)). We then compared the ZnF2-CGGA and the ZnF2-UU interaction surfaces. The latter interaction involves the side chains of residues His 1355 and Lys 1352 (His 11 and Lys 8 in this work) (Figure 2(a)), which are highly conserved across species (Supplementary Figure 4). The comparison shows that ZnF2 uses opposite surfaces and very different residues to bind the two RNAs, with both sets of residues being completely conserved in TUT4 and TUT7. In order to validate that U recognition is context-dependent, we tested the binding of the isolated hTUT4 ZnF2 to a UUUU RNA and observed no significant binding (Figure 2(b)). The use of two alternative surfaces for recognizing RNA points to the ZnF2 domain playing an additional and much broader role in the recognition of the RNA targets which is dependent on the overall context.

Interestingly, while we have no molecular data on the interaction of TUT4(7) (or other individual CCHC ZnFs) in complex with a G-rich sequence, high resolution structural data and biophysical data exists on the interaction of double CCHC ZnFs and short G-rich sequences. In particular, both high resolution structural information and CSP data are available on the interaction between the ZnF di-domain of hLin28 and the pre-Let-7 RNA target sequence. In this di-domain unit, the second ZnF interacts with a four nucleotide stretch within a larger RNA, with extensive contacts made with two central Gs whose individual mutation results in a drop in the affinity of the interaction [26]. We therefore compared the ZnF2-CGGA interaction surface mapped by our CSP data with the surface mediating the interaction between hLin28 ZnF2 and GG-containing 4-nucleotide RNA sequence, mapped using public domain data [26]. The comparison indicated that equivalent surfaces are used by hTUT4 ZnF2 and hLin28 ZnF2 (Figure 2(c)), although the residues mediating the interactions are not conserved between the two proteins, which implies the details of nucleobase recognition are different.

### ZnF2 is uncoupled from ZnF3 and acts independently in miRNA recognition

**Figure 3.**
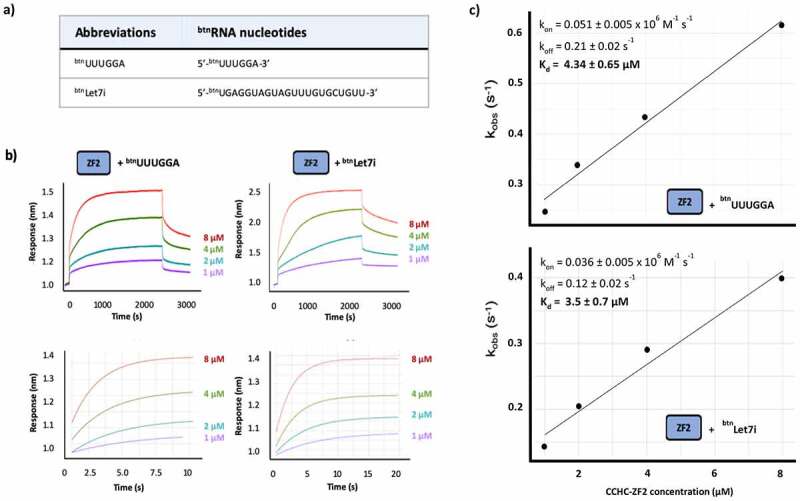
hTUT4 ZnF2-RNA binding using BioLayer Interferometry (BLI). a) Biotinylated RNAs used in the assays. b) Interferograms recorded using Streptavidin sensors coated with the biotinylated RNAs, in buffer and then exposed to increasing concentrations (1 µM, 2 µM, 4 µM and 8 µM) of hTUT4 ZnF2-3 domain. The interferograms are aligned using the buffer baseline. The baseline and the association and dissociation steps are displayed in the top panels. The region used to fit the association curves and to calculate k_obs _ is displayed in the bottom panel. c) Plots of k_obs_ at the different protein concentration. The plots were used to calculate k_on_ and k_off_, and from those K_d_, using the program Anabel

Two important questions are whether ZnF2 interaction with a short G-rich sequence recapitulates the one with a physiological RNA target(s) and, relatedly, whether the domain cooperates with hTUT4 ZnF1 and ZnF3 domains in this interaction. First, we explored the affinity and kinetics of ZnF2 interaction with the well-characterized Let7i miRNA and, compared them with those obtained for the interaction with a short RNA containing the SIA isolated sequence (Figure 3(a)) using Biolayer Interferometry (BLI). In our BLI assays, a biotinylated RNA is immobilized on a sensor and exposed to a series of protein concentrations (Figure 3(b)) and, in the short RNA, the SIA sequence has been distanced from the 5ʹ to prevent steric hindrance. Our data show that ZnF2 binds to both RNAs with affinity in the low micromolar range (~4 μM), and very similar kinetics (Figure 3(c)). Interestingly, the Let-7i sequence includes a GG di-nucleotide and a number of isolated Gs (Figure 3(a)), and mutation of the Gs in the miRNA resulted in the cumulative loss of binding and function [10,16].

Next, we want to understand whether ZnF2 and ZnF3 cooperate to create a stronger or more long-lasting interaction. CCHC ZnF domains have been shown to recognize RNA as a di-domain unit. However, the linker between the two domains is typically of a few amino acids. TUT4(7) ZnF2 is separated from the neighbouring ZnF3 domain by a long (~ 40 amino acids) linker. Further, although we have previously shown that ZnF3 selectively binds G-containing RNAs [25], the role of ZnF3 in promoting poly(U)ridylating pre-Let-7 miRNA is debated [21,22]. Whether the ZnF2 and ZnF3 domains cooperate in the recognition of the miRNA targets, and in general of TUT4 G-rich targets, is not known and requires direct investigation.

**Figure 4.**
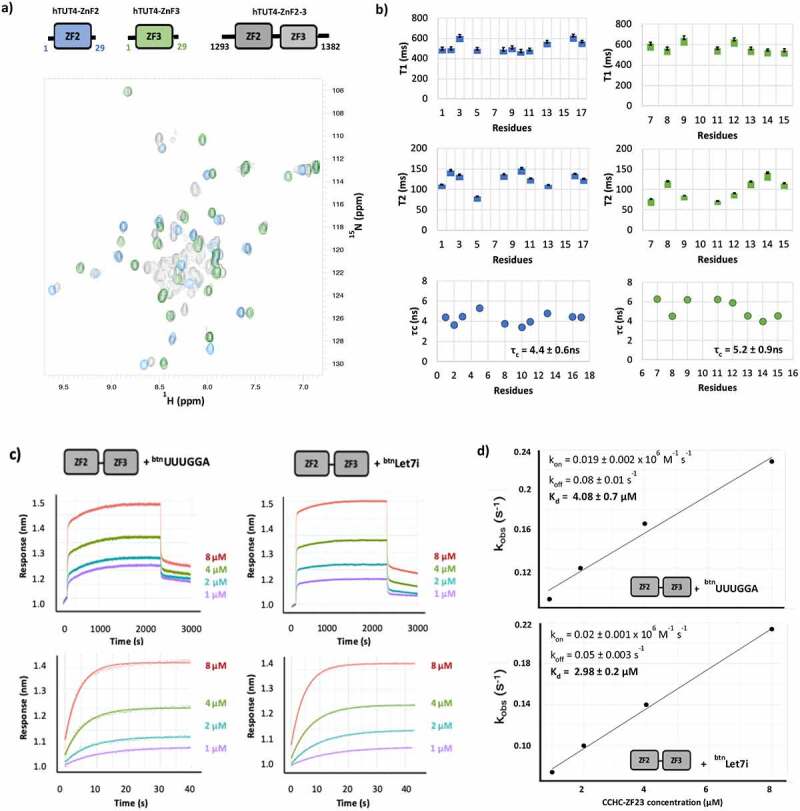
Structural and functional assessment of the coupling between hTUT4 ZnF2 and ZnF3 domains. a) Overlay of [^15^N ^1^H] HSQC spectra of ZnF2 (blue), ZnF3 (green) and ZnF2-3 di-domain (black). The organization of the two domains and the boundaries of the three constructs are displayed. b) ^15^N T1 and T_2_ and rotational correlation time (^τ^c) values of the ZnF2-3 amides are plotted along the protein sequence. Values for the ZnF2 and for the ZnF3 amides are reported in two separate plots, to better compare the two domains. Residues number refers to the sequence of each domain. c) Plots of K_obs_ at the different protein concentration. The plots were used to calculate ^k^on and ^k^off, and from those K_d_, using the program Anabel

In order to assess whether the ZnF2 and ZnF3 domains are interacting and operating as an RNA binding unit, we initially compared the fingerprint ^15^N-correlation NMR spectra of the ZnF2, ZnF3 and ZnF2-3 constructs (Figure 4(a)). Comparison of the spectra indicated that only minor differences are observed between the resonances of the isolated domains and the same resonances in two-domain construct indicating the two domains are unlikely to interact (Figure 4(a)). Consistently, the resonance attributable to the ZnF2-ZnF3 interdomain linker are in the random coil region, indicating the linker is unfolded. In order to validate this initial conclusion, backbone amide ^15^N T_1_ and T_2_ NMR relaxation experiments were recorded on the two-domain construct (Figure 4(b) and Supplementary Figure 5). The analysis of the relaxation series showed that ZnF2 and ZnF3 have rotational correlation times of τ_c_ = 4.2 ± 0.6ns, and τ_c_ = 5.2 ± 0.9ns, respectively. The domains’ correlation times confirm they are largely independent structural units that do not form a stable interaction. Next, we asked whether the two domains cooperate in binding the Let-7i miRNA. To answer this, we measured the strength and kinetics of the interaction between the ZnF2-3 di-domain unit and both the Let7i RNAs and the UUUGGA RNA, as a control where only one domain can bind (Figure 4(c-d)). We then compared the equilibrium and kinetic parameters with the ones obtained for the isolated ZnF2. Our experiments show that the ZnF2 and ZnF2-3 bind to both the miRNA and the short control RNA with very similar affinity and kinetics, although the ZnF2-3-miRNA complex has a slightly longer lifetime and slower association than the ZnF2 complex (~2-fold), and that binding of the two domains is, by and large, decoupled.

### hTUT4 ZnF1 has lost its ssRNA binding capability

**Figure 5.**
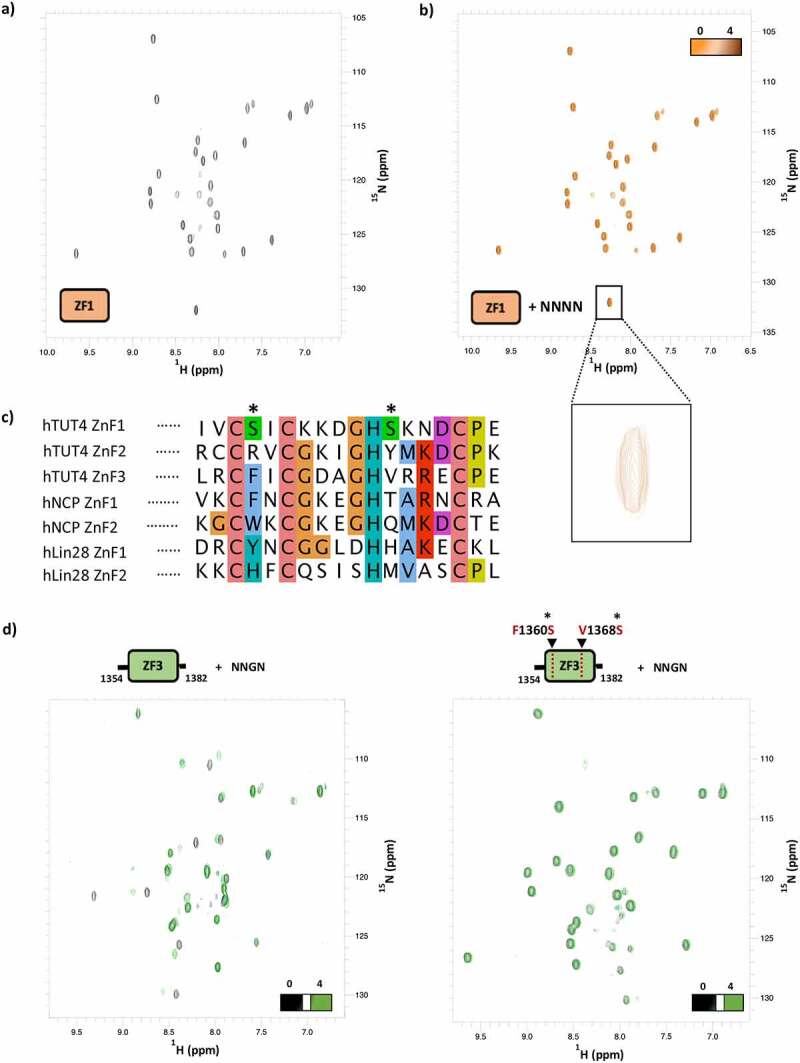
A) [^15^N ^1^H] HSQC spectra of CCHC-ZnF1. b) Overlay of the [^15^N ^1^H] HSQC spectra of ZnF1 free and with a 1:4 protein:NNNN RNA ratio. Zoom region of one ZnF1 residue to confirm spectral alignment and spectra quality. c) Sequence alignment of CCHC-type zinc fingers from hTUT4, HIV-1 nucleocapsid and hLin28 proteins. The ClustalX colour scheme is used to highlight conservation and residue type. In hTUT4 CCHC-ZnF1, two serines substitute bulky residues which are involved in RNA binding in the Lin-28 and Nucleocapside structures. d) Overlay of the [^15^N ^1^H] HSQC spectra of CCHC-ZnF3 (left) and CCHC-ZnF3 F1360S/V1368S mutant (right) free (black) and with NNGN RNA at a 1 to 4 ratio (green), which shows how mutating the two serines above impairs RNA binding

Finally, we wanted to assess whether the first CCHC ZnF1 of hTUT4 may also, in principle contribute to the recognition of the ssRNA regions/targets. The structural work by Daehnle and co-workers indicates this domain is likely to be decoupled by the NTD2 structure. We expressed and purified ZnF1, and recorded an NMR ^15^N-correlation spectrum that showed the domain’s amide resonance are well dispersed and the peaks have similar intensity. The number of peaks is consistent with the number of residues in the folded part of the domain confirming that ZnF1 is folded and assumes one stable conformation (Figure 5(a)).

Next, we explored ZnF1 RNA binding properties by titrating a NNNN RNA tetramer and monitoring spectra changes using NMR. We used a fully degenerate sequence in order to capture the sequence specificity of the domain which is unknown. The comparison of ^15^N-correlation experiments recorded at different titration points showed that ZnF1 amide resonances did not change their position in the presence of RNA, indicating the domain does not bind to RNA at a µM protein and RNA concentration (Figure 5(b)). This was surprising and in order to rationalize the lack of ZnF1 RNA binding activity, we compared the sequence of hTUT4 ZnF1 with the one of other two CCHC ZnF domains (ZnF2 and ZnF3) and with the sequences of the hLin28 and NCP double ZnFs [27], and mapped the residues important for the interaction in well-studied CCHC ZnF-RNA complexes. The sequence alignment highlights that a variable bulky hydrophobic residue that plays a key role in protein-RNA binding in the complexes above and is conserved in the ZnF2 and ZnF3 domains, is instead mutated to a Serine in ZnF1 (Figure 5(c)). A second, less conserved, bulky residue is also mutated to Serine. These differences provide a possible explanation for the loss of RNA binding by hTUT4 ZnF1. To validate our hypothesis, we generated a chimera in the ZnF3 domain where these residues are mutated to serine (F1360S/V1368S), to create a KH1 mimic. We then tested the mutant’s folding and RNA binding properties (Figure 5(d)). NMR ^15^N-correlation experiments showed that mutant ZnF3 maintains its fold but loses the capability to bind to a previously tested RNA oligo, NNGN [28].

## Discussion

TUT4, and the closely related TUT7 protein, are key factors in mammalian development that regulate the stability and processing of a range of RNA targets. The role of TUT4(7) in the uridylation of Let-7 miRNA precursor molecules has been studied in detail, but it is unclear how the protein(s) would recognize other ssRNA targets, including a set of mature ss miRNA molecules regulating immune response, growth hormone and development [16–18]. Here, we examine RNA binding by TUT4 ZnF domains and report that, while ZnF1 does not interact with ssRNA, ZnF2 recognizes a G-rich sequence using a surface alternative to the one mediating poly(U) recognition during the uridylation of Let-7 miRNA precursor. We then discuss the role of ZnF2 in the TUT4 interaction with the Let-7i miRNA, as a paradigm for the mature miRNA targets [21,26,29,30], and explore its relation with the adjacent ZnF3.

Our data show that hTUT4 ZnF1 and ZnF2 are independent folding units. However, while ZnF1 does not bind RNA, ZnF2 recognizes ssRNA with significant specificity. Importantly, ZnF2 does not recognize the U nucleobase that has been shown to make specific contacts with two amino acid side chains in a larger hTUT7 protein construct [21]. These contacts have been proposed to strengthen TUT4(7) processivity, leading to the synthesis of longer poly(U) chains [21]. Instead, our SIA assays and follow-up validation indicate that ZnF2 recognizes a GG di-nucleotide. A mechanistically important question is whether the interaction with the G-rich targets would prevent hTUT4 engaging the poly(U) tail of the pre-miRNA molecules, acting as a general molecular switch between different functions. While we do not have a definitive answer for the specific Let-7 pre-miRNA complex, the comparison of the two U- and GG-recognition surfaces indicates that the domain uses two non-overlapped sets of residues on opposite sides of ZnF2 to recognize the different sequences, and binding of one RNA does not imply release of the other. In addition, we do not observe binding of poly(U) sequences in our model system, even in the absence of other RNA, hinting that U recognition is dependent on a specific structural context. Regardless, our data indicate that ZnF2 uses a G-specific RNA-binding surface to recognize and recruit single stranded G-containing miRNA targets for TUT4 mono-uridylation and that the domain plays a role which is broader than the support of processive poly-uridylation in the pre-miRNA targets.

Many of TUT4(7) G-rich targets, including its miRNA targets, are regulated by adding a single Uridine [16]. In our model, the ZnF2 domain would help recognize those targets and recruit them to the polymerase. Therefore, we tested the interaction between ZnF2 and the best studied of these miRNA, Let-7i. Our data show that ZnF2 binds the full-length miRNA and the short SIA-derived G-rich target with very similar affinity and kinetics, indicating an interaction with a single site and an affinity which is not dependent on an avidity effect. This is confirmed by the association and dissociation kinetics which are similar to the ones we have observed for the interaction of single stranded, sequence specific RNA binding domains with the cognate RNAs [31,32]. This indicates that ZnF2 interacts with individual sites with low μM K_d_, and a higher affinity in the cell is likely reached by multiple synergistic interactions.

CCHC ZnF domains have been shown to recognize RNA sequences as part of di-ZnF structural units [26]. Although the linker between ZnF2 and ZnF3 is longer than the one observed in other di-domain units, ZnF2 could synergize with the downstream ZnF3, which we have shown also prefers G-rich sequences [25], to achieve the required affinity for the RNA target. Our chemical shift and relaxation NMR data, indicate that the ZnF2-3 linker is flexible and that the two domains do not interact in the free protein. Our data also show that the affinity and kinetics of the two-domain ZnF2-3 interaction with the miRNA are similar to the ones of the isolated ZnF2, indicating the presence of ZnF3 does not increases the affinity of ZnF2 for the physiological RNA target. It is worth mentioning that, we do not take this to imply that the two ZnFs are not binding simultaneously to the RNA molecule *in vivo*. Such binding may take place and, for example, help orient the RNA in the larger TUT4 complex. Rather, the decoupling of the two domains indicates that, contrary to what is observed for hLin28, target recognition by hTUT4 ZnFs is mediated by binding of individual domains that recognize very short RNA sequences. This is consistent with the overall molecular context being more important for hTUT4-RNA than for hLin28-RNA recognition, and with hTUT4 recognizing a broad range of RNA targets.

Our data on hTUT4 ZnF domains, reveal a context-dependent RNA recognition mode for hTUT4(7) ZnF2 and lead to a target selection model where the individual CCHC ZnF domains of hTUT4(7) can play multiple roles by stabilizing the nascent poly(U) chain, recruiting G-rich targets and possibly orienting these targets in the larger protein complex. How the latter would take place will require high-resolution structural information on a complex between the large hTUT4(7) CM module and a G-rich RNA target. Importantly, ZnF2 is not coupled to ZnF3 in binding the miRNA target, and the affinity and kinetics of the interaction are typical of individual protein domain binding short RNA sequences, rather than of a CCHC ZnF di-domain. It seems likely that, as for many other RNA binding proteins, higher affinity is reached by multiple inter-molecular interactions and, in this context, the role of ZnF1, which does not bind RNA and yet has a conserved interaction surface, may be to mediate some of the complex’s protein-protein contacts. Overall, our data paint a picture of complexity, where the different CCHC ZnF domains of TUT4(7) play very different roles and contribute to creating a network of interactions that define the multi-faceted function of this protein. Dissecting the interaction network using functional and molecular studies in different settings will represent a key step of understanding this global role.

## Material and methods

### Cloning, expression and purification of the protein constructs

hTUT4 zinc finger constructs CCHC‐ZnF1 (903‐931), CCHC‐ZnF2 (1293‐1321), CCHC‐ZnF2-3 (1293‐1382) and CCHC-ZnF3 (1354–1382) (PubMed Accession number NP_001009881, sequence in Supplementary Figure 6) were cloned from human cDNA into a pET‐M11 vector [33] to obtain protein constructs that include an N‐terminal hexa‐histidine tag (His_6_‐tag) followed by a cleavable tobacco etch virus (TEV) site and whose expression is under the control of a T7 promoter. Primers were designed to introduce the mutation F1360S/V1368V into CCHC-ZnF3. Point mutations were introduced into the construct by amplification of the plasmid using overlapping complementary primers with the mutation of interest inserted at the centre of the oligonucleotides. Following PCR amplification parent DNA was removed by DpnI digestion. The five constructs (ZnF1, ZnF2, ZnF2-3, ZnF3 and ZnF3 F1360S/V1368V) were expressed and purified using the protocols below.

In order to obtain ^15^N and, if required, ^13^C-labelled protein samples, the protein was expressed in M9 minimal media containing respectively (^15^NH_4_)_2_SO_4_ as the only nitrogen source and ^13^C_6_‐D‐glucose as the only carbon source. *E. coli* BL21(DE3) cells were transformed with the pET-M11 relevant plasmid and grown overnight at 37°C. The overnight culture was diluted to an OD_600_ of 0.1 in fresh media and cells were grown to an OD_600_ of 0.6 before the temperature was reduced to 22°C and protein expression induced with 0.5 mM IPTG. Protein expression was overnight at 22°C, and cells were harvested by centrifugation and stored at ‐80°C.

Cells were sonicated in denaturing buffer pH 8.0 (100 mM NaH_2_PO_4_, 10 mM Tris‐HCl pH 8.0, 8 M urea) and the lysate was centrifuged at ~7700 x *g* for 30 minutes. The supernatant was loaded on a Ni-NTA column and purified by immobilized metal ion affinity chromatography (IMAC). In more detail, 4 ml of Ni‐NTA resin per litre of cell culture was packed into a gravity flow column and equilibrated with 10 column volumes (CV) denaturing buffer. The clarified cell lysate was loaded onto the column and the flow through was collected and reloaded onto the column to enhance binding of the expressed protein. The protein was eluted with denaturing buffer pH 4.5 (100 mM NaH_2_PO_4_, 10 mM Tris‐HCl pH 4.5, 8 M urea). The eluted protein was dialysed against 4 litres of equilibration buffer (10 mM Tris pH 8.0, 10 mM Imidazole, 200 mM NaCl, 2 mM β‐mercaptoethanol, 10 μM ZnCl_2_) to reduce the urea content in the buffer. TEV protease was then added to a final concentration of 2.5 μM to cleave the His_6_‐tag. The reaction mixture was dialysed for a further 16 hours for cleavage to take place. Both protease and cleaved tag were separated from the protein by a reverse IMAC column (Ni‐NTA). The cleavage reaction mixture was loaded into a gravity flow column packed with Ni‐NTA resin equilibrated with 10 mM Tris pH 8.0, 10 mM Imidazole, 200 mM NaCl, 2 mM β‐mercaptoethanol, 10 μM ZnCl_2_. The flow through was collected and reloaded onto the column for a second time and the flow through from the second column was concentrated using a Vivaspin concentrator and further purified using a Hiload 16/60 Superdex 75 gel filtration column. Fractions containing pure protein as assessed by SDS-PAGE were pooled and concentrated before being dialysed into a final buffer of 10 mM Tris‐HCl pH 7.4, 100 mM NaCl, 0.5 mM TCEP and 10 μM ZnCl_2_ and concentrated in a Vivaspin to the final storage concentration. Protein concentration was determined from the absorbance at 280 nm and the molecular weights of the different constructs confirmed by mass spectrometry.

### RNA sample preparation

RNA oligonucleotides (CGGA, CUGA, CGUA, UUUU, ^btn^UUUGGA, ^btn^UGAGGUAGUAGUUUGUGCUGUU and ^btn^UCACCUACUACUUUCUCCUCUU) were purchased from Horizon Discovery, and deprotected as advised. RNA concentration was determined from the 260 nm absorbance of the sample.

### NMR protein backbone assignment experiments

Labelled (^15^N or ^13^C^15^N) hTUT4 samples were prepared as described and concentrated to ~200 μM. NMR experiments were recorded at 25°C using a Bruker Avance NMR spectrometer operating at 600 MHz ^1^H frequency. Measurements were made in 90% H_2_O/10% D_2_O. A ^13^C^15^N labelled hTUT4 CCHC‐ZnF2 sample was used to acquire a HNCA, HN(CO)CA [34], HNCACB [35], HN(CO)CACB [36] and HNCO at 10 mM Tris‐HCl pH 7.4, 100 mM NaCl, 0.5 mM TCEP and 10 μM ZnCl_2_ buffer conditions. Data were processed using Topspin 3.7 and assignment of protein resonances was done on CcpNmr analysis [37].

### NMR relaxation measurements

Relaxation experiments [38] were recorded on a ^15^N‐labelled hTUT4 ZnF2-3 sample to obtain T_1_ and T_2_ values. Experiments were performed on a Varian Inova NMR spectrometer operating at 800 MHz ^1^H frequency. 20, 200, 400, 700, 1000, 1500 ms delays and 10, 20, 40, 70, 100, 150 ms delays were used for T_1_ and T_2_ measurements, respectively. Standard relaxation values were determined for each residue by fitting an exponential decay to the peak volume over the course of the data collected. Residues were excluded where overlap in the signals prevented accurate measurement on peak volume.

The hTUT4 ZnF2 peaks used in the di-domain relaxation to calculate the correlation time in that domain are C1295, R1296, V1297, G1299, G1302, H1303, Y1304, M1305, D1307, K1310 and R1311 (C3, R4, V5, G7, G10, H11, Y12, M13, D15, K18 and K19 in this work). In the hTUT4 ZnF3 case the residues used were C1360, F1361, I1362, G1364, D1365, A1366, G1367 and H1368 (C7, F8, I9, G11, D12, A13, G14, H15, in this work).

### NMR titration experiments

NMR RNA titrations were performed in 10 mM Tris‐HCl pH 7.4, 100 mM NaCl, 0.5 mM TCEP and 10 μM ZnCl_2_ buffer conditions at 25°C. ^15^N HSQC NMR spectra were recorded during the titration of a 50 μM protein sample with increasing at each concentration of the different RNAs (at protein RNA ratios 1 to 0, 0.5, 1, 2, 4, 6, 8, 10, 12). Data were processed using Topspin 3.7 (Bruker) and a value for the ^1^H and ^15^N Chemical Shift changes was calculated for each residue applying the following equation: ($\Delta \delta =\sqrt{\left({\left(\Delta \delta_{NH}\right)}^{2}+{\left(\Delta \delta_{N}*0.15\right)}^{2}\right)}$ to the shifts in the two dimension.

The K_d_ for the hTUT4 ZnF2-CGGA interaction were calculated in the CcpNmr analysis program using the A(B + x-sqrt((B + x)^2-4x) equation. K_d_ were calculated for individual residues and then merged to obtain the global Kdan average of all individual K_d_ per residue [39].

### NMR scaffold independent analysis

70 μM hTUT4 ZnF2 stock was prepared in 100 mM NaCl, 10 mM Tris pH 7.4 buffer containing 0.5 mM TCEP, 10 μM ZnCl_2_, sodium azide and RNasin Plus. This was aliquoted out into 180 μM samples and RNA pools added where required at a protein to RNA ratio of 1 to 4. Samples were transferred to 3 mm NMR tubes and placed in a rack. Samples were automatically loaded into the spectrometer using the Bruker Sample Jet and ^1^H‐^15^N SOFAST‐HMQC spectra were recorded for each of the samples. NMR data were recorded on a Bruker Avance NMR spectrometer at 700 MHz. Spectra were processed using NmrPipe and data analysed using the weighted average chemical shift changes (for H^N^ and N) with each RNA pool for a subset of shifting residues being measured and normalized with respect to the highest shift. The average normalized value across the subset of peaks is taken to give a comparative score of binding preference.

### BioLayer Interferometry (BLI) measurements

BLI experiments were performed in 10 mM Tris pH 7.4, 100 mM NaCl, 0.5 mM TCEP, 10 μM ZnCl_2_, 0.5 mg/ml bovine serum albumin (BSA) on an Octet Red 96 instruments (ForteBio, Inc. Menlo Park, CA) operating at 25°C. 5ʹ-Biotinylated 5ʹ-^btn^UUUGGA-3ʹ and Let7i miRNA variations (5ʹ-^btn^UGAGGUAGUAGUUUGUGCUGUU-3ʹ, 5ʹ-^btn^UCACCUACUACUUUCUCCUCUU-3ʹ) (1 ng μL−1 solutions) were immobilized on streptavidin-coated biosensors and incubated with varying concentrations of hTUT4 ZnF2 and ZnF2-3 (1–8 μM). Equilibrium and kinetic parameters were extracted by the analysis of the observed association rate constant versus ligand concentration using the program Anabel, as described [40].

### Sequence alignment and structures used

All sequence alignments were carried out using the programs T-Coffe [41] and EMBOSS Needle [42] with default settings. Figures were generated using ClustalX [43]. The hTUT7 coordinates used in our image building derive from the 5W0M PDB file, while the coordinates of the hLin28 ZnFs were extracted from the PDB file 2LI8.

## Supplementary Material

Supplemental Material

## Data Sharing Statement

The plasmids expressing the constructs discussed in this study will be made available by the authors upon request. The resonances assignment of hTUT4 ZnF2 protein will be available the BMRB database (https://bmrb.io/) upon publication of the manuscript.
