# Supplementary material for: The distinct RNA-interaction modes of a small ZnF domain underlay TUT4(7) diverse action in miRNA regulation
Source: RNA Biol. 2021 Nov 1;18(Suppl 2):770–81. doi: 10.1080/15476286.2021.1991169 (PMC8782169; doi:10.1080/15476286.2021.1991169)
Supplement: Supplemental Material [file KRNB_A_1991169_SM3565.pdf]

Supplementary Figure 1.

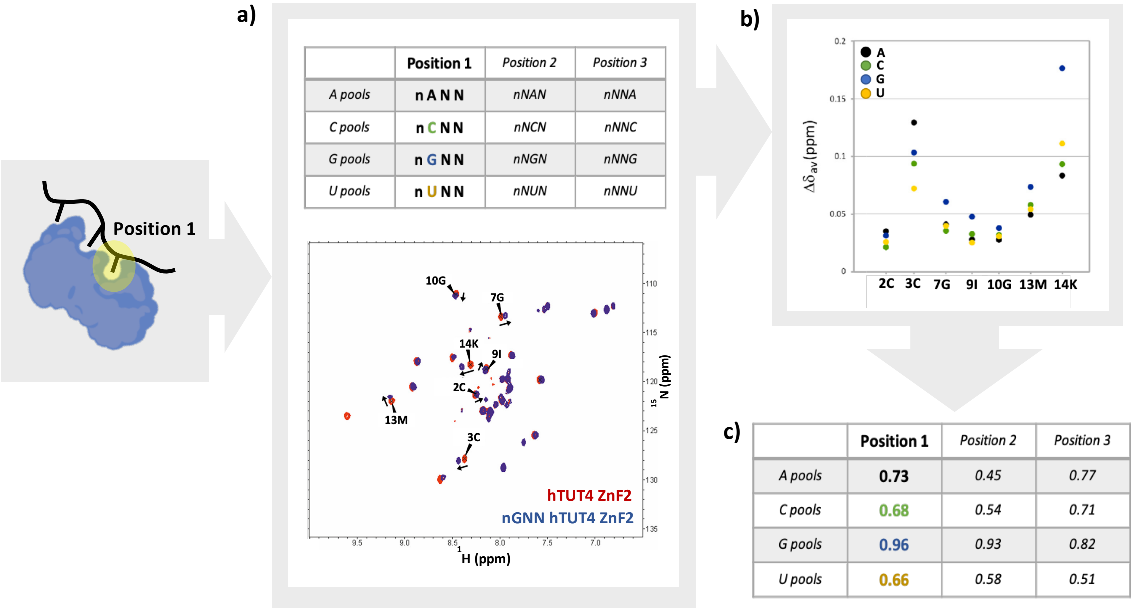

Supplementary Figure 1. *Scaffold Independent Analysis (SIA) workflow. The data and workflow for the determination of the nucleobase preference of hTUT4 ZnF2 for position 1 of the bound sequence are shown as an example.* ***a)*** *Four quasi-randomized RNA pools differing in the nucleobase to be examined (either A, C, G or U) were added to the protein to a 1:4 ratio, and* ^{1}H*-*^{15}N *HSQC spectra were recorded for the free and bound proteins. Chemical shift changes of shifting peaks in the fast exchange regime were measured.* ***b)*** *The chemical shifts changes (*^{1}H *and* ^{15}N *weighted average) of each peak in the titration with the four nucleotides were plotted as shown here. They were then normalised with respect to the highest shift value so that each peak contributes equally to the output.* ***c)*** *Normalised values are then averaged over the set of residues to give the final scores reported here.*

Supplementary Figure 2.

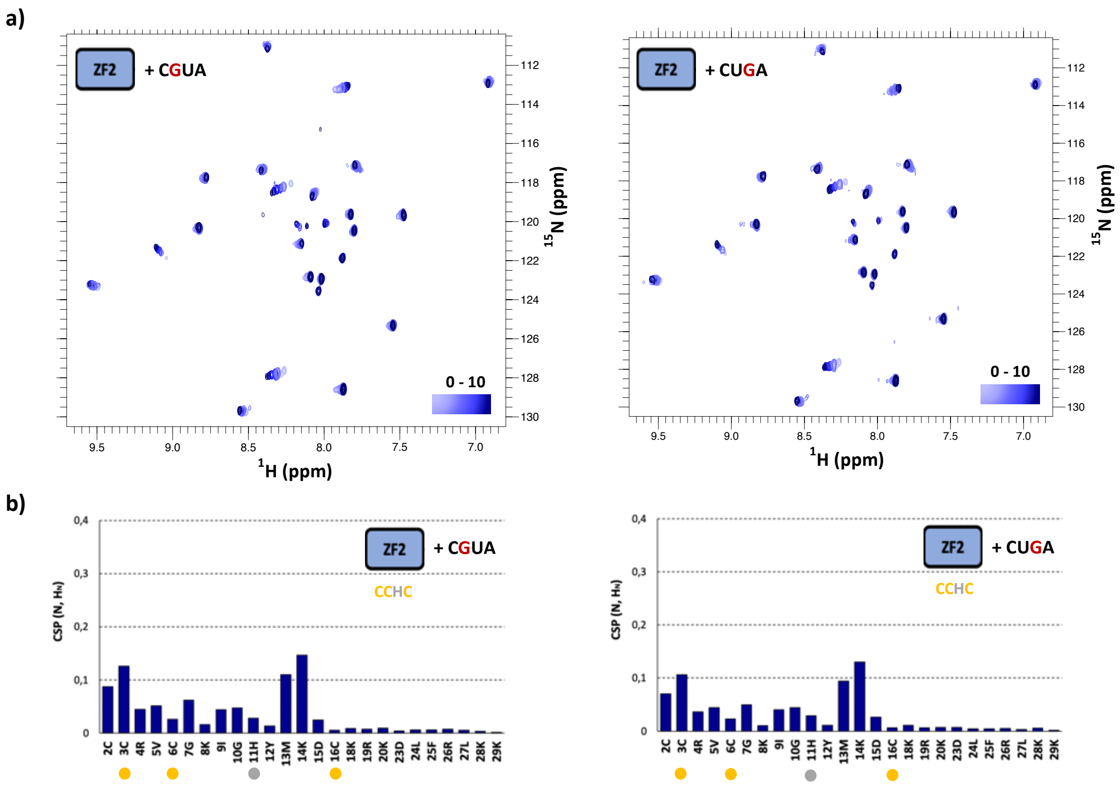

***Supplementary Figure 2**. **a)** Overlay of [^{15}N-^{1}H] HSQC spectra of hTUT4 ZnF2 with CGUA (left) and CUGA (right) at protein to RNA ratios from 1 to 0 (light blue) to 1 to 10 (dark blue). **b)** The chemical shift changes (^{1}H and ^{15}N weighted average) at a protein to RNA ratio of 1 to 10 is plotted along the protein sequence. The residues coordinating the Zinc ion (CCHC) are plotted in yellow (C) and grey (H).*

Supplementary Figure 3.

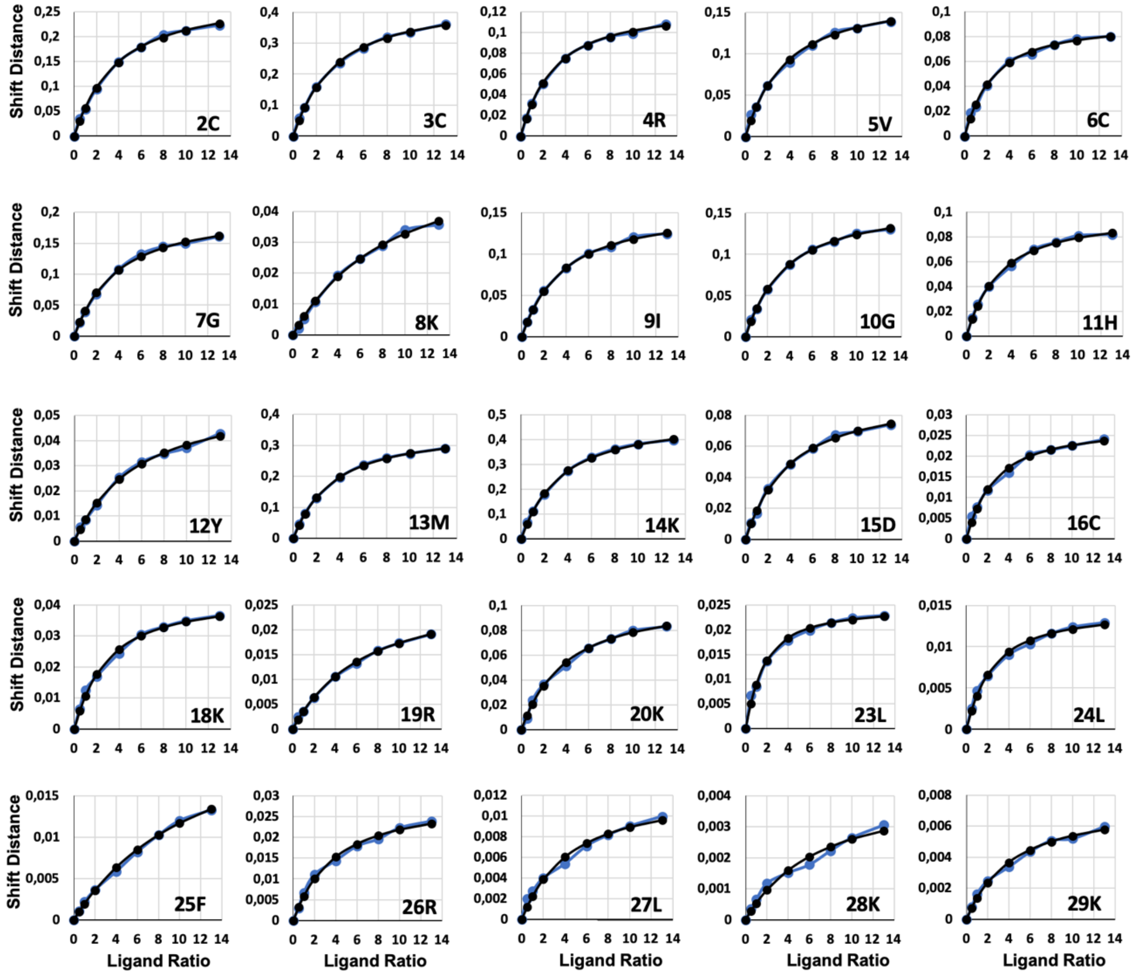

Supplementary Figure 3. *Isotherms obtained from CSP data (^1H and ^{15}N weighted average) obtained during a titration of hTUT4 ZnF2 with CGGA RNA. Data points (blue) and calculated fitted values (black) are plotted for the individual residues. Fitting was performed using CcpNmr and the equation A(B+x-sqrt((B+x)^2-4x). The K_d values and errors reported in the manuscript are an average of the ones obtained for the individual residues.*

Supplementary Figure 4.

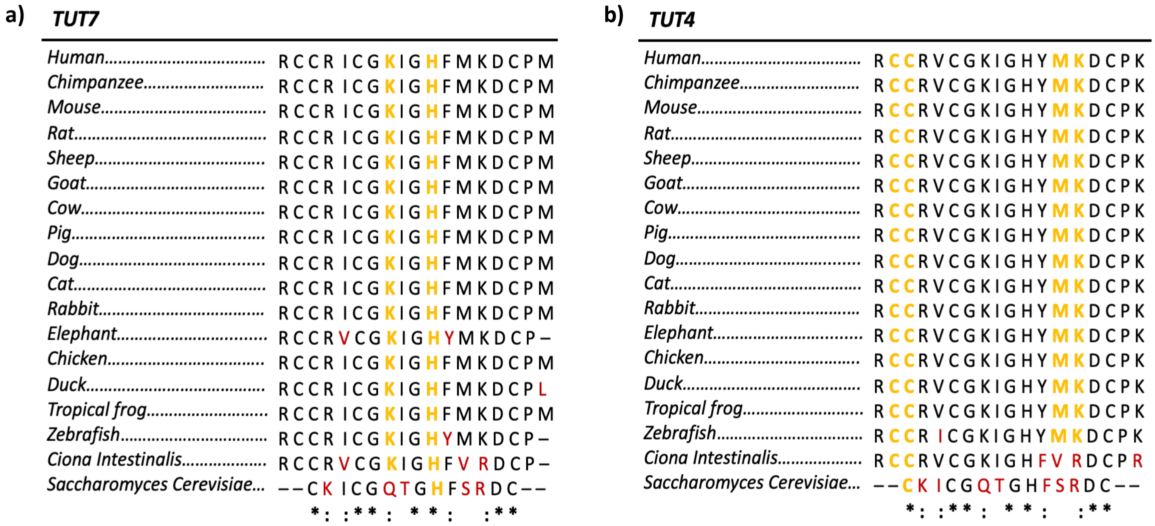

***Supplementary Figure 4**. Sequence alignment of hTUT4 and hTUT7 proteins ZnF2 domain(s). **a)** Sequence alignment of hTUT7 ZnF2 across species. The residues involved in the ZnF2-UU interaction are highlighted in yellow. The residues which are not conserved across species are highlighted in red. **b)** Sequence alignment of hTUT4 ZnF2 across species. The residues involved in the ZnF2-CGGA interaction are coloured in yellow. The residues which are not conserved are coloured in red. Asterisks (*) and colons (:) indicates fully conserved and highly conserved residues respectively.*

Supplementary Figure 5.

hTUT4 ZnF2	T1 ± error (ms)	T2 ± error (ms)
1	498.9 ± 14.1	111.6 ± 0.9
2	502.0 ± 14.5	146.4 ± 2.0
3	625.9 ± 13.0	135.9 ± 1.5
5	496.2 ± 12.0	83.1 ± 0.7
8	491.0 ± 16.9	137.1 ± 1.5
9	511.4 ± 14.2	-
10	474.9 ± 16.4	151.4 ± 1.9
11	489.9 ± 13.3	126.8 ± 1.3
13	567.2 ± 11.9	111.2 ± 1.0
16	628.0 ± 13.2	138.0 ± 1.5
17	568.6 ± 12.4	126.3 ± 1.2

hTUT4 ZnF3	T1 ± error (ms)	T2 ± error (ms)
7	609.9 ± 15.1	76.7 ± 0.5
8	561.9 ± 13.6	120.7 ± 1.2
9	666.3 ± 17.1	85.1 ± 0.5
11	565.5 ± 11.8	71.9 ± 0.5
12	651.6 ± 13.7	91.4 ± 0.8
13	562.1 ± 12.5	119.7 ± 1.2
14	545.8 ± 9.3	141.7 ± 2.0
15	544.8 ± 14.2	115.5 ± 1.0

Supplementary Figure 5*. ^{15}N T_1 and T_2 NMR relaxation values and fitting errors for the backbone amide of hTUT4 ZnF2 and ZnF3 within the ZnF2-3 construct.*

Supplementary Figure 6.

```
   1 meesktlkse nhepkknvic eeskavqvig nqtlkarndk svkeienssp nrnsskknkq
  61 ndiciektev ksckvnaanl pgpkdlglvl rdqshckakk fpnspvkaek atisqaksek
 121 atslqakaek spkspnsvka ekassyqmks ekvpsspaea ekgpslllkd mrqktelqqi
 181 gkkipssfts vdkvnieavg gekcalqnsp rsqkqqtctd ntgdsddsas giedvsddls
 241 kmkndesnke nssemdylen atvidesalt peqrlglkqa eerlerdhif rlekrspeyt
 301 ncrylcklcl ihieniqgah khikekrhkk nilekqeese lrslpppspa hlaalsvavi
 361 elakehgitd ddlrvrqeiv eemskvittf lpecslrlyg ssltrfalks sdvnidikfp
 421 pkmnhpdlli kvlgilkknv lyvdvesdfh akvpvvvcrd rksgllcrvs agndmacltt
 481 dlltalgkie pvfiplvlaf rywaklcyid sqtdggipsy cfalmvmffl qqrkppllpc
 541 llgswiegfd pkrmddfqlk giveekfvkw ecnsssatek nsiaeenkak adqpkddtkk
 601 tetdnqsnam kekhgkspla letpnrvslg qlwlellkfy tldfaleeyv icvriqdilt
 661 renknwpkrr iaiedpfsvk rnvarslnsq lvyeyvverf raayryfacp qtkggnkstv
 721 dfkkrekgki snkkpvksnn matngcillg ettekinaer eqpvqcdemd ctsqrciidn
 781 nnllvneldf adhgqdsssl stsksseiep kldkkqddla psetclkkel sqcncidlsk
 841 spdpdkstgt dcrsnletes shqsvctdts atscnckate dasdlndddn lptqelyyvf
 901 dkfiltsgkp ptivcsickk dghskndcpe dfrkidlkpl ppmtnrfrei ldlvckrcfd
 961 elsppcseqh nreqiligle kfiqkeydek arlclfgssk ngfgfrdsdl dicmtleghe
1021 naeklnckei ienlakilkr hpglrnilpi ttakvpivkf ehrrsglegd islyntlaqh
1081 ntrmlatyaa idprvqylgy tmkvfakrcd igdasrgsls syayilmvly flqqrkppvi
1141 pvlqeifdgk qipqrmvdgw nafffdktee lkkrlpslgk nteslgelwl gllrfyteef
1201 dfkeyvisir qkklltfek qwtskciaie dpfdlnhnlg agvsrkmtnf imkafingrk
1261 lfgtpfypli greaeyffds rvltdgelap ndrccrvcgk ighymkdcpk rkssllfrlk
1321 kkdseeekeg neeekdsrdv ldprdlhdtr dfrdprdlrc ficgdaghvr recpevklar
1381 qrnssvaaaq lvrnlvnaqq vagsaqqqgd qsirtrqsse csespsyspq pqpfpqnssq
1441 saaitqpssq pgsqpklgpp qqgaqpphqv qmplynfpqs ppaqyspmhn mgllpmhplq
1501 ipapswpihg pvihsapgsa psniglndps iifaqpaarp vaipntshdg hwprtvapns
1561 lvnsgavgns epgfrgltpp ipwehaprph fplvpaswpy glhqnfmhqg narfqpnkpf
1621 ytqdrcatrr crercphppr gnvse
```

Supplementary Figure 6*. Amino acid sequence of Homo Sapiens Terminal Uridyl Transferase Isoform A. PubMed Accession number NP_001009881.*
